# Association between vertebral artery hypoplasia and posterior circulation stroke

**DOI:** 10.1186/s12883-016-0644-x

**Published:** 2016-07-26

**Authors:** Virginija Gaigalaite, Augenijus Vilimas, Violeta Ozeraitiene, Jurate Dementaviciene, Ricardas Janilionis, Danute Kalibatiene, Saulius Rocka

**Affiliations:** 1 Department of Neurology and Neurosurgery, Vilnius University, Vilnius, Lithuania; 2 Faculty of Medicine, Vilnius University, M. K. Ciurlionio g. 21, Vilnius, LT-03101 Lithuania; 3 Department of Fundamentals of Internal Medicine, Vilnius University, Vilnius, Lithuania; 4 Department of Radiology, Nuclear Medicine and Physics of Medicine, Vilnius University, Vilnius, Lithuania

**Keywords:** Vertebral artery, Hypoplasia, Stroke, Occlusion, Age, Gender, Anthropometry

## Abstract

**Background:**

The clinical significance of vertebral artery (VA) hypoplasia is under discussion. The aim of this retrospective study is to evaluate a hypothesis of a possible causal link between VA hypoplasia (VAH) and the incidence of posterior circulation stroke (PCS) or TIA depending on the degree of VAH  and vascular risk factors.

**Methods:**

A total of 367 symptomatic (PCS or TIA) and 742 asymptomatic subjects, were selected to participate in the study. The extracranial arteries were examined by ultrasound. VAH was defined as VA diameter in entire course <3 mm, although different degrees of VAH were examined. All the symptomatic patients underwent MRI or CT and MRA or CTA. The study assessed all the subjects in terms of their age, gender, co-risk factors (hypertension, hyperlipidemia, diabetes mellitus, peripheral arterial diseases, atrial fibrillation, myocardial infarction), as well as height of 180 healthy volunteers.

**Results:**

VAH, regardless of the degree of severity, was more frequent in patients with non-cardioembolic PCS or TIA rather than in asymptomatic patients. The increasing degree of hypoplasia in patients under 65 years of age was a predictor of PCS/TIA, OR = 1.8, 95 % CI: 1.3-2.5; *p* < 0.001. In subjects older than 65 years of age, this association failed. Only in patients aged under 50, VAH was significantly more frequent in the TIA group rather than in the PCS group (68.2 % and 50 %, respectively; *p* = 0.047). The optimal VA diameter cutoff point separating PCS/TIA and asymptomatic group was 2.7 mm. This value may vary in different populations, because VA diameter showed a significant dependence on sex as well as anthropometric parameters (height). With the increasing degree of VAH, the likelihood of the occurrence of the distal VA part stenosis/occlusion was growing (OR = 1.6, 95 % CI: 1.2-2.1; *p* = 0.002). The distal VA stenosis/occlusion was likely to occur where the VA diameter was <2.2 mm.

**Conclusions:**

The impact of the VAH on PCS/TIA and its pathogenetic mechanism was significantly influenced by age. The cutoff point of VA diameter, affecting the occurrence of PCS in different populations may vary because VA diameter depends on gender and anthropometric parameters (especially height).

## Background

Vertebral artery hypoplasia (VAH) is a relatively frequent anatomical variation of vertebral arteries (VA). Due to its high prevalence, the clinical significance of VAH has been underestimated. However, ipsilateral VAH is commonly observed in patients with posterior circulation stroke (PCS), suggesting that VAH confers an increased probability of ischemic stroke [1–6]. To date, the definition of VAH remains inconclusive. The diameters between 2 and 3 mm, as well as an asymmetry ratio threshold >1:1.7 have been used to describe VAH but there is no consensus on this value [1, 7]. A retrospective analysis of patients showed that VAH defined as VA of diameter <2 mm is more frequent in patients with PCS rather than in asymptomatic patients or in patients with other locations of cerebral infarctions [1, 3]. Another analysis showed that VAH defined as VA of diameter <2.5 mm is more frequent in patients with PCS rather than in patients with other locations of cerebral infarctions [1, 4]. Some studies indicate that VAH defined as VA of diameter <3 mm is also more common in patients with PCS [1, 2].

It is not clear which diameter of VA can be considered as a risk factor for PCS, or is this diameter universal for different populations? Nor it is clear if VAH is an independent risk factor for PCS, or it is a risk factor for PCS only after contributed additional atherosclerotic risk factors, and what type of an etiopathogenetic mechanism of PCS it is. For further research, it is important to determine an optimal clinically significant cutoff value of VA diameter for VAH definition in the relevant population, and thereby to identify a high-risk PCS group among patients with VAH. As a commonly used test in carrying out a research, an ultrasound examination is considered to be not only inexpensive, harmless and suitable to a large group of people, but can also be repeated several times. Until recently, the determination of VAH cutoff point value has been based on significant hemodynamic changes detected by color-coded duplex ultrasonography (2.5 mm detected by a decreased net vertebral flow volume <100 mL/min [8] or 2.2 mm detected by an increase of ipsilateral flow resistance (resistance index >0.75) [9]. However, even if the net vertebral flow volume is decreased, it is not clear whether this is a risk factor of PCS. Overall, more detailed studies are necessary to determine the pathophysiological and causative relationship between VAH and PCS. Is the relationship between the degree of VAH and PCS likely to depend on the patient’s age, sex or other risk factors?

One of the possible mechanisms of PCS at the presence of VAH is a local hypoperfusion in the proximal part of the vertebrobasilar territory (of basilar artery and VA branch) [1, 7]. The authors [7] noted that VAH defined as VA of diameter <2 mm (V4 segment) lead to regional hypoperfusion in the dependent posterior inferior cerebellar artery (PICA) territory in 42 % of patients, as identified by the whole-brain CT perfusion. Future studies should not only address whether the patients with local hypoperfusion in the proximal part of vertebrobasilar territory indeed have an increased risk for PCS but also they need to determine the cutoff point of VA diameter influencing such hypoperfusion. In addition to the local hypoperfusion, some authors argue that patients with VAH are more susceptible to stenosis or occlusion of the distal VA [2–4], i.e. VAH increases the risk of ischemic stroke of atherosclerotic origin. This evidence was considered to relate to the slow blood flow in VA which might increase the susceptibility to thrombosis and poor clearance of thrombi resulting in stenosis of the distal artery [2–4].

The aim of this retrospective study is to evaluate a hypothesis of a possible causal link between VAH and the incidence of posterior circulation stroke or TIA depending on the degree of VAH and the risk factors – the patients’ age, gender other adjacent risk factors.

To achieve this and to verify the agreement of clinical data with possible PCS/TIA etiologies due to local hypoperfusion or atherosclerosis, we sought to (1) assess the frequency of VAH in patients with PCS or/and TIA as compared to patients without a cerebrovascular disease (TIA/stroke); (2) identify the optimal cut off diameter for VAH which discriminates individuals with PCS/TIA from asymptomatic (without a cerebrovascular disease) ones; (3) to compare the frequency of coexistence vascular risk factors in symptomatic (PCS/TIA) and asymptomatic individuals with VAH; (4) identify the individuals with VAH for which the hypothesis that the increasing VAH degree i.e. progressive blood flow reduction through VA progressively increases the risk of PCS/TIA; (5) identify the individuals for which the hypothesis that severe VAH increased the risk of distal VA part occlusion and therefore PCS/TIA may be related to large vessel atherosclerosis.

## Methods

The present study was based on a sample of 367 symptomatic patients who were treated in the Republican Vilnius University Hospital from 2013 through 2014 (249 with first-ever PCS and 118 with TIA), and 742 asymptomatic subjects, i.e. people without cerebrovascular disease (TIA or stroke) history before and at the time of the study enrolment. The extracranial carotid and vertebral arteries of all the subjects involved in the study were examined by ultrasound.

The duplex ultrasonographic examination of the extracranial arteries (vertebral and carotid) was performed by using the 7.5 MHz linear array transducer of Aloka Prosound F 75 ultrasound system. Since we wanted to analyze the relation between PCS/TIA and different degrees of VA hypoplasia (from slight to severe) we have chosen the broadest definition of VAH, i.e. we defined VA as hypoplastic when VA diameter in the entire course was less than 3 mm [2, 3]. Although the VA was examined by ultrasound in the entire extracranial part (for PCS/TIA patients they were additionally examined by CTA/MRA in entire course), similar to [3, 4, 6] we have chosen to present the distance between the internal layers of VA from the level V2 (the portion in the vertebral columns) because other levels, including V3 (after exit from the C2 transverse foramen), and V1 (proximal to entry into the transverse foramen) have more artifacts than V2 and the distance measurements may be less accurate. Hemodynamic characteristic of blood flow were registered in levels V1 and V2.

However, we examined different degrees of VAH: VA in diameter of <2.2 mm, VA in diameter of 2.2-2.4 mm, VA in diameter of 2.5-2.6 and VA in diameter of 2.7-2.9 mm. The study excluded the patients with the internal carotid artery (ICA) stenosis >50 % or occlusion as well as the patients whose extracranial arteries’ assessment by ultrasound has failed. MRI or CT and magnetic resonance angiography (MRA) or computed tomography angiography (CTA) were performed on all the symptomatic patients. The report assesses intracranial VA and other vessels of circle of Willis’ (hypoplasia, aplasia, stenosis >50 %, occlusion) and the location of ischemic lesions. In patients with atherosclerotic stenosis or occlusion in the VA (defined by MRA or CTA) the stenosis degree of the distal vertebral artery (V4) was defined as “occlusion”, “stenosis” (stenosis > 50 %) and “normal” (normal or stenosis < 50 %). The study excluded the patients who did not undergo MRI/CT and MRA/CTA investigation, nor those who were diagnosed with intracerebral hemorrhage, whose stroke was localized in the anterior circulation area and those who were diagnosed with the carotid artery or with the middle cerebral artery or with the anterior cerebral artery stenosis >50 % or occlusion. The subtypes of stroke were classified into categories based on etiology using TOAST classification.

We assessed all the subjects in terms of demographic indicators (age, gender), anthropometric indicators (height of 180 healthy volunteers was registered) and stroke risk factors (hypertension, hyperlipidemia, diabetes mellitus, peripheral arterial disease, atrial fibrillation, myocardial infarction).

### Statistics

The *Chi* square independence (χ2) test was applied in carrying out the comparison between the categorical variables and the determination of the odds ratio, while Fisher’s exact test was used in the case of a small sample size. Continuous variables meeting assumptions of normality were analyzed using t-tests for independent groups. Mann-Whitney U test was used for any continuous variables not meeting the normality assumptions or ordinal variables. The VA diameter distribution curve is shown in accordance with the kernel density graph and the cumulative distribution curve. VA distribution curves were compared with each other by using non-parametric tests (Mann-Whitney U test was applied when comparing two samples or Kruskal -Wallis test – when comparing more than two samples). The VA diameter’s cutoff point separating the symptomatic and asymptomatic subjects and those with VA stenosis/occlusion and without stenosis was determined in carrying out the ROC analysis. The significance of the association between the continuous values was found by calculating the Pearson’s correlation coefficient (r). The logistic regression analysis was used to find the independent variables that predict the binary outcome. The independent predictors such as age, sex, vascular risk factors, VA hypoplasia (different degrees) were included in the logistic regression model. The chosen significance level α = 0.05.

## Results

### VA diameter in the asymptomatic group (*n* = 742)

**Fig. 1:**
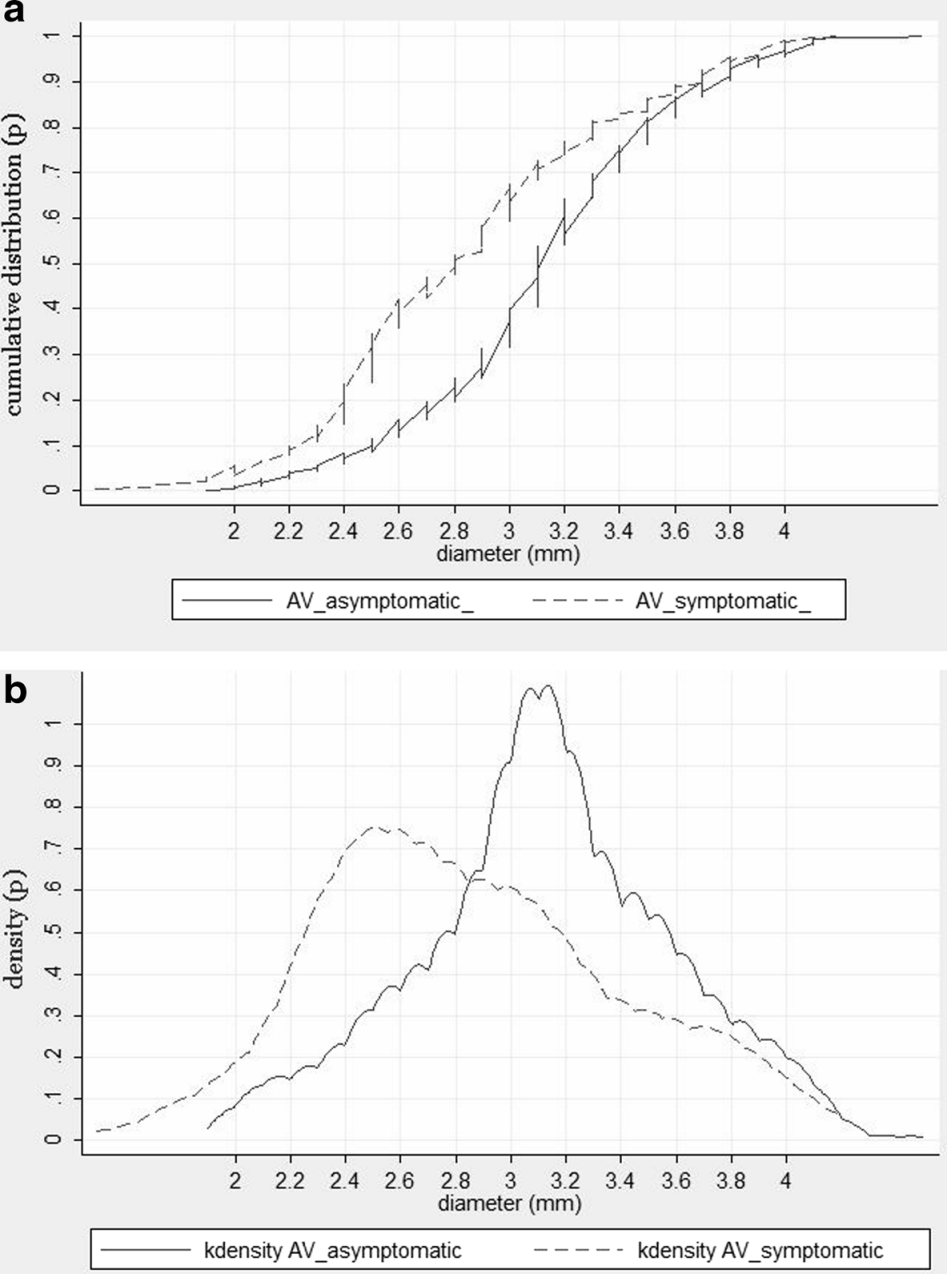
**a**. Comparative cumulative non-dominant VA diameter distribution curves in asymptomatic and symptomatic subjects. **b**. Kernel density of VAH diameter in symptomatic and asymptomatic patients

VAH (VA diameter is defined as <3 mm) of a varying degree was diagnosed in 31.3 % of the population. The non-dominant VA diameter <2.2 mm was observed in 2.6 % of the subjects, VA diameter <2.5 mm – in 8.3 % of the subjects, VA diameter <2.7 mm – in 15.7 % of the subjects. A more detailed cumulative distribution curve of the non-dominant VA diameter is shown in Fig. 1a. The left VA was more than twice more frequently (65.8 % and 31.7 % respectively) larger than the right one, while both the diameters were equal in 2.5 % of the cases. The VAH was also more common on the right than on the left. The right VAH was observed in 20.3 % of the cases, the left - 9.8 %, on both sides – 1.2 %. VAH was more common in women (33 %) rather than in men (23.5 %), *p* = 0.012. Also, VA in males was found to be wider than in females. Net vertebral artery diameter (left + right) was 7.1 ± 0.05 mm and 6.7 ± 0.04 mm, respectively, *p* < 0.001, dominant VA diameter 3.9 ± 0.03 mm and 3.6 ± 0.02 mm, respectively, *p* < 0.001, non-dominant VA diameter 3.2 ± 0.02 mm and 3 ± 0.01 mm, respectively, *p* = 0.032.

Results in Table 1 shows a weak but statistically significant correlation between the dominant VA diameter and height, between both net vertebral artery diameter and height and between the left VA diameter and height in 180 healthy volunteers whose height was registered. It is noteworthy that the average of both net VA diameter in subjects taller than 180 cm was 7.2 ± 0.09 mm, while in those shorter than 160 cm – 6.6 ± 0.2 mm, *p* = 0.038. The average of the dominant VA diameter in subjects taller than 180 cm was 3.9 ± 0.06 mm, while in those shorter than 160 cm – 3.5 ± 0.1 mm, *p* = 0.017.

**Table 1:** Correlation between VA diameter and height (*n* = 180)

	Height	
	r	p
Dominant VA diameter	0.25	0.001
Left VA diameter	0.2	0.01
Non-dominant VA diameter	0.15	>0.05
Right VA diameter	0.18	>0.05
Net vertebral artery diameter	0.22	0.001

### The association between VAH and PCS/TIA

VAH was more frequent in patients who suffered from PCS or TIA rather than in asymptomatic subjects (58.3 % and 31.3 %, respectively, *p* < 0.01). It is notable, that in patients with stroke localized in other than posterior territory, i.e. anterior circulation stroke (*n* = 128) the frequency of VAH (30.4 %) did not differ from asymptomatic group (*p* > 0.05). The net vertebral artery diameter was larger in the asymptomatic group rather than in the group of sufferers from PCS or TIA (6.8 ± 0.03 mm and 6.3 ± 0.08 mm, respectively, *p* < 0.001). The non-dominant VA diameter was smaller in the group with PCS/TIA rather than in the asymptomatic group (2.8 ± 0.03 mm and 3.1 ± 0.02 mm, *p* < 0.001). The dominant VA diameter was not significantly different in both groups (3.8 ± 0.01 mm in asymptomatic and 3.7 ± 0.04 mm in symptomatic subjects, *p* > 0.05). More detailed differences of the non-dominant VA diameter distribution in symptomatic and asymptomatic patients are provided in Fig. 1a (cumulative curve) and Fig. 1b (kernel density graph).

**Fig. 2:**
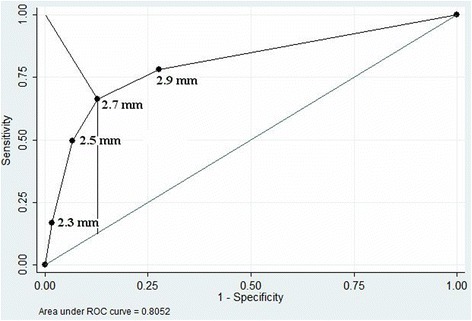
ROC curve of non-dominant VA diameter in asymptomatic and symptomatic patients

The ROC analysis (see Fig. 2) showed that the optimal cutoff point of VA diameter separating the PCS/TIA and the asymptomatic group is 2.7 mm (Youden’s index (the maximum vertical distance or the difference between the ROC curve and the diagonal), or the closest point in the upper left corner), so VA diameter <2.7 mm can be assessed as a risk factor for PCS/TIA.

By means of the logistic regression analysis it was determined that with the increasing level of VA hypoplasia, i.e. with the gradual decrease of VA diameter (>3 mm, 2.7-2.9 mm, 2.5-2.6 mm, 2.2-2.4 mm and <2.2 mm), the likelihood of developing PCS/TIA is increasing (OR = 1.5, 95 % CI: 1.1-1.9; *p* = 0.01). The analysis of the PCS/TIA dependency on the growth of the degree of VA hypoplasia in different age categories (<45 years, 45-54 years, 55-64 years, 65 -74 years and >75 years) shows that it is not homogeneous. The increasing degree of hypoplasia in subjects aged under 65, is a predictor of PCS/TIA, OR = 1.8, 95 % CI: 1.3-2.5; *p* < 0.001. Meanwhile, the category of subjects older than 65 years showed no clear association between the increase in the degree of hypoplasia and PCS/TIA risk (OR = 1.2, 95 % CI: 0.7-1.5; *p* = 0.06). In summary, although VA diameter <2.7 mm can be assessed as a risk factor for PCS or TIA, the clear association between the decreasing VA diameter and PCS/TIA risk was only in younger subjects aged under 65.

### The comparison of symptomatic and asymptomatic subjects with VAH risk factors

Table 2 shows that symptomatic patients were more likely to develop arterial hypertension rather than asymptomatic (73.8 % and 65.1 %, respectively, *p* = 0.045), among them men (57.5 % and 42.7 %, *p* = 0.002) prevailed in asymptomatic subjects. Other risk factors did not show any statistically significant difference. However, it should be noted that patients with first-ever PCS were older than patients with a milder degree of circulatory deficiency, i.e. TIA (62.2 ± 1.5 years and 56.3 ± 1.1 years, respectively, *p* < 0.001). However, regardless of the age, more than one vascular risk factors were more frequently observed in patients with a history of PCS rather than TIA (45.9 % and 27 %, respectively, *p* = 0.01).

**Table 2:** Comparison of symptomatic and asymptomatic subjects with VAH risk factors

	Asymptomatic (*n* = 232)	Symptomatic (*n* = 214)	p
Age (years)	56.9 ± 1.1	57.3 ± 2.2	NS
Gender (Male/Female) (%)	42.7/57.3	57.5/42.5	0.002
Arterial hypertension (%)	65.1	73.8	0.045
Diabetes (%)	6.9	6.5	NS
Coronary artery disease without myocardial infarction (%)	5.6	7.9	NS
Myocardial infarction (%)	3.0	3.3	NS
Hypercholesterolemia (%)	47.8	51.4	NS
Peripheral vascular diseases (%)	1.3	1.9	NS
Atrial fibrillation (%)	0.9	4.2	NS

### VAH and the etiology of stroke

Table 3 presents the prevalence of VAH among patients with different etiology of PCS and TIA. Table 3 shows that VAH is observed as significantly least frequent (33.3 %) in patients who suffered from cardioembolic strokes as compared with stroke of other etiology (*p* = 0.026). The difference between the proportion of VAH among cardioembolic stroke patients (33.3 %) and asymptomatic subjects (31.3 %) was not significantly different (*p* > 0.05). The VAH ratio between TIA and PCS patients was not significantly different (61 % and 57 %, *p* > 0.05, respectively). However, in different age categories the difference between the prevalence of VAH in patients with TIA and in patients with PCS was not homogeneous. Only in the age category of patients younger than 50 years (*n* = 116), VAH was significantly more frequent in patients with milder circulatory disorder, i.e., TIA than PCS (68.2 % and 50 %, respectively, OR = 1.32, 95 % CI: 0.7-2.3; *p* = 0.047). In patients older than 50 years (*n* = 251), no statistically significant difference was observed (51.9 % and 58.8 %, respectively, *p* > 0.05).

**Table 3:** Prevalence of VAH among patients with different etiology of PCS and TIA

	Prevalence of VAH	p	p
TIA (*n* = 118) (%)	61		NS
Cerebral ischemic stroke (*n* = 249) (%)	57		
▪ Large vessel disease (*n* = 60) (%)	58.3	*p* = 0.026	
▪ Small vessel disease (*n* = 50) (%)	58		
▪ Undetermined (*n* = 106) (%)	63.2		
▪ Cardioembolic (*n* = 33) (%)	33.3		

### The relationship between VA hypoplasia and distal VA stenosis > 50 % or occlusion in symptomatic patients

**Fig. 3:**
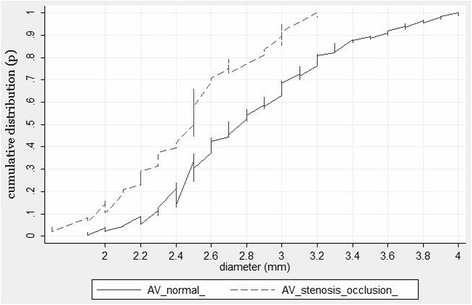
Cumulative VA diameter distribution curves in cases with stenosed/occluded and normal VA

**Fig. 4:**
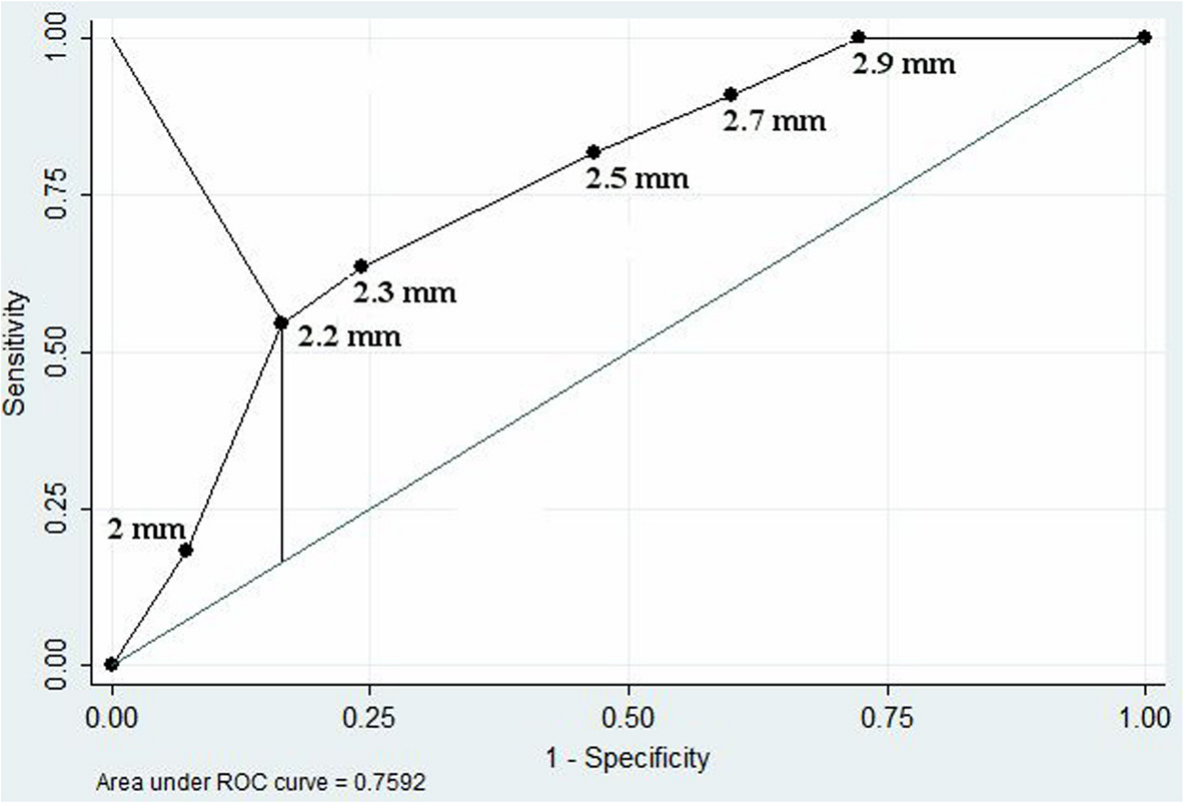
ROC curve of VA diameter separating VA stenosis/occlusion from normal VA

**Fig. 5:**
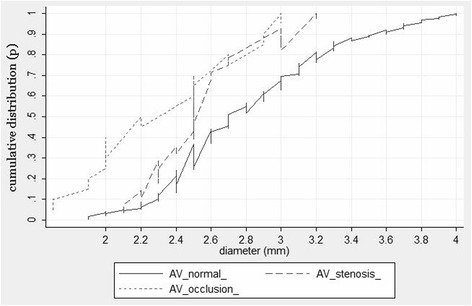
Cumulative VA diameter distribution curves in cases with stenosed, occluded and normal VA

The distal stenosis >50 % or occlusion of VA of normal diameter (not less than 3 mm) was observed in 5.2 % of the cases. Meanwhile, the VAH stenosis or occlusion was observed even in 16.8 % of the cases, *p* = 0.006. In the cases with VA stenosis/occlusion, the VA diameter was less than in cases with normal VA (corresponding mean 2.46 ± 0.07 mm and 3.05 ± 0.05 mm, *p* = 0.01). More detailed VA diameter distribution differences in the presence or absence of VA stenosis/occlusion are presented in Fig. 3. These distribution curves show a statistically significant difference (*p* = 0.006, Mann-Whitney U test). The logistic regression analysis determined that with the increasing level of VA hypoplasia, i.e. with the decrease of VA diameter (>3 mm, 2.7-2.9 mm, 2.5-2.6 mm, 2.2-2.4 mm and <2.2 mm) the likelihood of the occurrence of the VA stenosis/occlusion is growing (OR = 1.6, 95 % CI: 1.2-2.1; *p* = 0.002). Among VA with diameter <2.2 mm, the number of stenosed/occluded VA amounted to 22.9 %, while in those with VA diameter >3 mm, the number of stenosed/occluded VA was only 5.6 %, *p* = 0.015. The ROC (see Fig. 4) analysis determined the optimal cutoff point of the VA diameter separating VA stenosis/occlusion from normal VA which is equal to 2.2 mm (Youden’s index and the point closest to the upper-left corner). Thus, VA diameter less than 2.2 mm can be considered a risk factor of a severe distal VA stenosis or occlusion. Fig. 5 shows the comparative cumulative VA diameter distribution curves in cases with stenosed, occluded and normal VA. These were statistically significant differences between the VA diameter distribution curves (*p* < 0.001, Kruskal-Wallis test). The VA diameter in the cases with an occluded VA (*n* = 18) was the smallest compared to those with stenosed VA (*n* = 18) or normal VA (2.27 ± 0.05 mm, 2.5 ± 0.09 mm and 3.05 ± 0.05 mm, respectively, *p* = 0.03). Due to a small number of patients with occluded distal VA (*n* = 18), a statistical analysis of the impact triggered by the decreasing VA diameter on VA occlusion is not possible.

### Risk factors for VAH stenosis or occlusion

Table 4 shows that the incidence of arterial hypertension (94.4 % and 70.2 %, respectively, *p* = 0.002) and coronary heart disease (19.4 % and 5.6 %, respectively, *p* = 0.005) was higher in patients with distal VAH stenosis >50 % or occlusion. Also, they were older (64.3 ± 2.2 years and 55.9 ± 1 year, respectively, *p* < 0.001). A high degree of stenosis or occlusion was observed even in 28.2 % of people older than 65 years and only in 10.3 % of those younger than 65 years, *p* = 0.01.

**Table 4:** Comparison of risk factors in patients with VAH stenosis/occlusion and those with normal VAH

	Absence of stenosis (%) (*N* = 178)	Presence of stenosis/occlusion (%) (*N* = 36)	p
Age (years)	55.9 ± 1.0	64.3 ± 2.2	<0.001
Gender (Male/Female) (%)	56.8/43.2	61.1/ 38.9	NS
Arterial hypertension (%)	70.2	94.4	0.002
Diabetes (%)	6.7	5.6	NS
Coronary artery disease without myocardial infarction (%)	5.6	19.4	0.005
Myocardial infarction (%)	2.8	5.6	NS
Hypercholesterolemia (%)	50	58.3	NS
Peripheral vascular disease (%)	1.1	5.6	NS
Atrial fibrillation (%)	3.4	8.3	NS

## Discussion

Our results show that the occurrence of VAH is more common in patients with PCS or TIA of non-cardioembolic origin rather than in asymptomatic patients. According to [3] the luminal narrowing of the VAH might make it less feasible for cardiogenic emboli to pass through it. On the other hand, the findings obtained by diverse authors indicate that both PCS risk and outcome depend on the impairment of the posterior circulation hemodynamics and the compensating potential of collateral circulation [10, 11]. Similarly to other authors [12], our findings show that ischemic strokes of non-cardioembolic origin were most commonly (more than 60 % of cases) localized in the proximal part of the vertebrobasilar system, i.e. in the brainstem or cerebellum, while the strokes of cardioembolic origin – in the distal part of the vertebrobasilar system. It has been proven that in terms of both VAH, total or local VA pathology, hypoperfusion is local and is observed only in the proximal part of the vertebrobasilar system [2, 3, 7], i.e. the compensatory properties of the blood flow circulation are reduced only in these zones. Thus, if due to VAH, the blood circulation may be aggravated in the proximal part of the vertebrobasilar system. It can increase the risk of PCS or TIA of the appropriate etiology, being localized in the proximal part of vertebrobasilar system, i.e., PCS/TIA of non-cardioembolic origin.

The analysis of PCS risk factors showed the dependence of the association between VAH and PCS/TIA on age. Only in patients younger than 65 years, the increasing degree of VAH, i.e. the increasing local hypoperfusion, is related to the increasing risk of PCS/TIA. However, in older subjects with more vascular risk factors such obvious connection was not observed. In addition, in people younger than 50 years, VAH was associated with a milder circulatory deficiency – TIA rather than PCS. This finding is likely to be associated with the fact that in younger age, due to the frequent absence of risk factors, the prevalence of a hypoperfusal circulatory disorder mechanism may be observed and the evident association between the degree of reduction in the blood flow through VA, increasing the local hypoperfusion in the proximal part of the vertebrobasilar system and the risk of PCS/TIA is logical. In older people, the atherosclerosis related risk factors were the main contributors which led to the severity of circulatory disorders, rather than the decreased blood flow through VAH. Naturally, when the hypoperfusal circulatory disorder mechanism is contributed by the atherosclerotic circulatory disorder mechanism which often affects a number of blood vessels (not only single VA) by reducing their lumens, a correlation between the increasing degree of VAH and the risk of PCS/TIA becomes less pronounced. However, there is a need for a more explicit and more extensive research into different age groups, with varying degrees of circulatory problems seeking to find the effects of adjacent risk factors on the association of PCS/TIA and varying degrees of VAH as well as on their impact on the etiology of stroke in patients with VAH.

The distal VAH part tends to occlude or a high degree of stenosis is developed there, i.e. the risk of atherosclerotic process in hypoplastic arteries is higher rather than in arteries of normal diameter [2–4]. The smaller the VA diameter is, the higher the risk of stenosis/occlusion is observed. According to our data, the optimal cut-off diameter of VAH for VA stenosis/occlusion is VA diameter <2.2 mm. The total number of 22.9 % stenosed/occluded VA patients were observed among those with PCS and with VA diameter of <2.2 mm, while the number of those with stenosed/occluded VA among subjects with the diameter >3 mm was only 5.6 %, *p* = 0.015. The authors [3] describe thrombosis of the distal part of VA even in a wider range of patients with PCS and VAH (defined as VA diameter <2 mm in V4 segment) – in 89.1 %. The VAH tendency to occlude is interpreted taking into account the fact that due to reduced blood flow through VA, the clearance of the occurring clots deteriorates which leads to stenosis or occlusion of the distal part of VA [3]. In addition, the risk of stenosis/occlusion in patients with VAH is increased by vascular risk factors. In accordance with our data, a group of patients with stenosed/occluded distal VAH showed higher risk factors which reduce the elasticity of blood vessels and increase the number of peripheral resistance: they were older or showed a more frequent incidence of arterial hypertension.

Many authors agree on the fact that a small diameter VA is likely to occlude. However, the mechanism of stroke remains unclear when occlusion occurs in VA of a small diameter through which the blood flow is generally low (<30 ml/min [8]), therefore, upon its occlusion, only a mild decrease in the net flow volume is observed. Can a slight decrease in the local hypoperfusion be considered the PCS cause, or maybe PCS is an outcome of thrombosis in situ? The VAH atherosclerotic process tends to be not localized but spreads to other intracranial blood vessels and small blood vessels in the brain become a reason of PCS of mixed etiology. The analysis of the risk factors of PCS patients with stenosed/occluded VAH confirms that they involve a higher number of vascular risk factors, increasing both the total hypoperfusion and triggering the progression of the atherosclerotic process, i.e. they are running the risk of stroke of mixed etiology. However, a more detailed research is necessary into the differences of VAH effects on the brain blood flow observed in young and old age, collateral circulation and the circle of Willis characteristics at the presence of VAH, the spread of atherosclerotic process to other intracranial arteries at the presence of VAH stenosis/occlusion, the impact of the disease of small blood vessels on VAH thrombosis and PCS.

VAH diameter <2.7 mm is the optimal VA diameter observed in our subjects to increase the risk of PCS. However, we believe that this rate may vary in examining different populations, because the VAH optimal diameter value depends on the difference between the non-dominant VA diameter distribution between asymptomatic people and those suffering from PCS/TIA. Moreover, it is influenced by the difference between the corresponding net vertebral artery diameter, i.e. the corresponding compensating potential. Striking differences were observed while comparing the scholarly data on the prevalence of VAH of the same degree. For example, the VAH defined as diameter <2 mm was found by some authors [13] only in 1.9 % of the population, by other authors [9] – 11.6 %, while in accordance to some of the authors [3] – even in 26.5 %. Even greater differences are identified while comparing the data, provided by various authors on the patients suffered from PCS. A high degree of VAH (defined as diameter <2 mm) proportion in PCS patients ranged from 10-20 % [4, 6] PCS patients to nearly half of PCS patients (45.6 %) [3]. A lack of universal methodology aimed at measuring VA diameter explains only part of the possible differences observed in the prevalence of VAH in the population. The distribution of VA diameter depends on the indicators of the population under examination such as gender, height, comorbidities. VAH is more common [5, 14, 15] in patients with migraine with aura or those suffering from vestibular neuronitis rather than in those without these diseases). Therefore, the data provided by individual authors may be comparable, if we examine the prevalence of same degree of VAH, the studies are carried out according to the same research methodology, the categories of population under analysis are similar in terms of their demographic, anthropometric characteristics and coexisting diseases. However, the information about the differences between the categories of population under analysis is usually not known. Very large differences in the prevalence of VAH are observed when comparing the prevalence of VA hypoplasia between the Asian (China, Korea) population, the majority of whom are of short height, and the European population, who are taller [1, 3]. Apart from the mentioned reasons, the prevalence of VAH among PCS can differ in different populations due to other reasons impacting the prevalence of VAH, i.e. diverse etiology of stroke, different age of patients, etc.

## Conclusions

The impact of the vertebral artery hypoplasia on PCS/TIA and its pathogenetic mechanism was significantly influenced by age. The cut-off point of VA diameter, affecting the occurrence of PCS in different populations may vary because VA diameter depends on gender and anthropometric parameters (especially height).

## Abbreviations

CT, computed tomography; CTA, computed tomography angiography; MRA, magnetic resonance angiography; MRI, magnetic resonance imaging; PCS, posterior circulation stroke; PICA, posterior inferior cerebellar artery; TIA, transient ischemic attack; VA, vertebral artery; VAH, vertebral artery hypoplasia/hypoplastic vertebral artery
